# Non-islet cell tumor hypoglycemia caused by breast tumor: A case report

**DOI:** 10.1097/MD.0000000000027889

**Published:** 2021-12-03

**Authors:** Zhibing Zhou, Wensong Wei, Jianhong Tu, Qihua Jiang

**Affiliations:** The Third Department of Mammary Gland, Third Hospital of Nanchang, Nanchang, China.

**Keywords:** insulin-like growth factor II, non-islet cell tumor hypoglycemia, phyllodes tumor of the breast

## Abstract

**Introduction:**

Non-islet cell tumor hypoglycemia (NICTH) generally refers to hypoglycemia caused by tumors other than islet cell tumors. Although hypoglycemia is a common clinical emergency, NICTH rarely occurs in patients with breast cancer.

**Patient concerns:**

A 47-year-old woman presented with repeated hypoglycemia hypoglycemia caused by a lobulated breast tumor.

**Diagnoses:**

Hypoglycemic symptoms occurred many times during fasting and in the early morning. Insulin and C-peptide levels were decreased; insulin-like growth factor (IGF)-II: IGF-I was greater than 10. Postoperative pathology revealed a lobulated tumor in the breast. After excluding other causes of hypoglycemia, the patient was diagnosed with NICTH due to breast cancer.

**Interventions:**

Total mastectomy of right breast was performed.

**Outcomes:**

After 3 years of follow-up, hypoglycemia did not recur.

**Conclusion:**

Patients with breast cancer may experience recurrent hypoglycemia. After exclusion of insulinomatous and pancreatic origin of hypoglycemia, the possibility of NICTH should be considered, and surgical resection of the primary tumor should be performed as soon as possible.

## 1 Introduction

Hypoglycemia is a common clinical condition observed during diabetes treatment and endocrine disorders. Abnormal glucose metabolism is caused by an increase in hypoglycemic substances, such as insulin, or a decrease in glucose levels. Hypoglycemia, which is not caused by islet cell tumors, is known as non-islet cell tumor hypoglycemia, which is relatively rare. Here, we report a case of hypoglycemia caused by a phyllodes tumor of the breast.

## 2 Case report

This study was approved by the Medical Ethics Committee of The Third Hospital of Nanchang, and written informed consent was obtained from the patient for publication of the case details.

### 2.1 Clinical data

A 47-year-old woman presented with an acute painful face without obvious induction of a one-day duration. She was unconscious, comatose, no nausea and vomiting, no limb convulsions, and no incontinence of urine and faces. She went to the local hospital for an emergency blood glucose check of 1.71 mmol/L and was relieved after intravenous glucose infusion. Physical examination of the right breast showed a 15 cm × 13 cm hard mass, that was suspected as right breast tumor. The patient had no history of diabetes or hypertension, and had no family history of cancer. The patient was transferred to our hospital for further treatment, of the breast mass for nearly 2 year duration and paroxysmal hypoglycemia of 1 day duration

Physical examination revealed bilateral breast asymmetry; right breast palpation revealed large mass, approximately 20 × 20 cm in size, solid, with unclear boundary, and thickened veins on the skin of the breast. There was no obvious abnormal mass in the left breast and no bilateral axillary enlarged lymph nodes.

### 2.2 Laboratory examination revealed

Three episodes of hypoglycemia: 6 mmol/L, 1. 7 mmol/L and 2. 3 mmol/L respectively. Oral 75 g glucose tolerance and insulin release test result: blood glucose 0 minutes 1.6 mmol/L, 30 minutes 5.4 mmol/L, 60 minutes 11.8 mmol/L, 120 minutes 7.1 mmol/L, 180 minutes 3.3 mmol /L, 240 minutes 1.7 mmol/L; Insulin 0 minutes 0.48 mU/L, 30 minutes 14.15 mU/L, 60 minutes 10.36 mU/L, 120 minutes 2.68 mU/L, 180 minutes 0.69 mU/L, 240 minutes 0.48 mU/L; C-peptide levels: 0 minutes 0.26 ng /mL, 30 minutes 0.19 ng /mL, 60 minutes 0.03 ng /mL, 120 minutes 0.04 ng/mL, 180 minutes 0.02 ng/mL, 240 minutes 0.00 ng/mL. Fasting insulin- levels: was 0.48 mu/L (normal value 3.00–25.00 mu/L), fasting anti insulin antibody was 4.69% (normal value 0.00%–5.00%), glycosylated hemoglobin was 5.0% (normal value 4.0%–6.5%), cortisol was 69.3 nmol/L (normal value 69.0–345.0 nmol/L) in the afternoon and 400.0 nmol/L (normal value 138.0–690.0 nmol/L) in the morning, ACTH level was 36.50 ng/L (normal value 7.60–76.00 ng/L), Growth hormone level was 0.123 μg/L (normal value <8 μg/L). Blood routine, urine and stool routine, liver and kidney function, electrolytes, myocardial enzymes, hypertension and tumor were normal. In addition, blood samples were collected, centrifuged and frozen at − 20°C, following the procedure of immunoradiometric test kit provided by DSL company and Insulin-like growth factor(IGF)-I level was 110.6 μg/L (normal value 104.4∼203.0 μg/L), IGF-II level was 2973.4 μg/L (normal value 520.58∼2745.58 μg/L).

### 2.3 Supplementary examination

**Figure 1:**
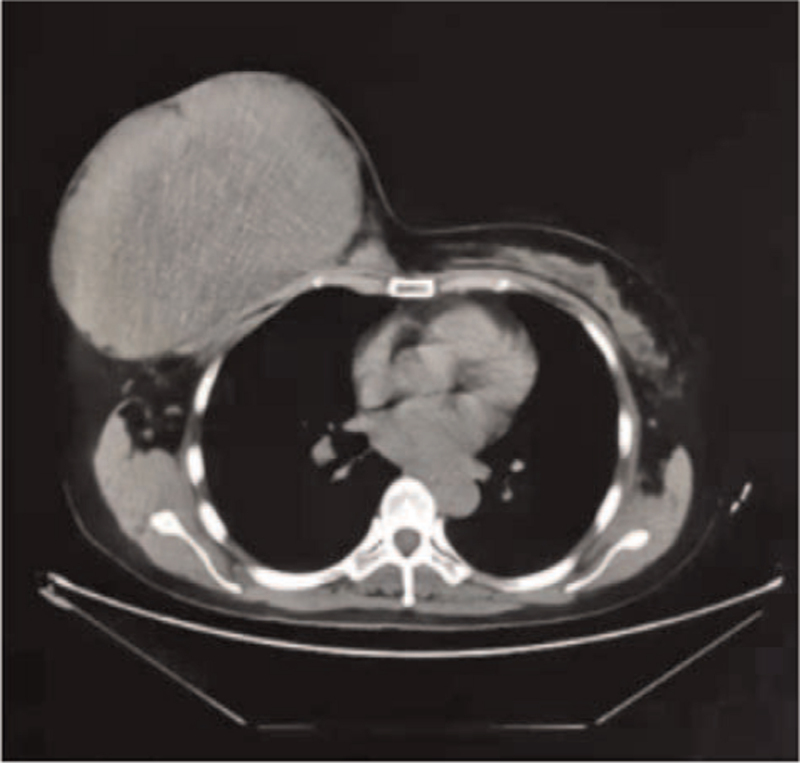
Chest CT showed a huge mass in the right breast.

Color Doppler ultrasonography of the breast and axillary lymph nodes showed a hypoechoic mass in the right breast with a regular shape, clear margin, uneven internal echo, and calcification. Color Doppler flow imaging findings: Blood flow signal grade III. In view of the abundant blood flow signals, it was suggested to further check the exclusion of Ca, acrbi-rads-us4. Chest computed tomography revealed giant space-occupying lesions in the right breast and no abnormalities in the lung, mediastinum, upper abdomen, or bilateral adrenal glands (Fig. 1). Pituitary magnetic resonance imaging showed no abnormalities, and the bone scan showed1. No obvious signs of malignant bone lesions; 2. The right 2 to 7 anterior costal area had a large mass of dense foci. The color Doppler ultrasound of liver, gallbladder, spleen, pancreas and urinary system were normal. Gynecological color Doppler ultrasound showed cervical cyst (1.0 ∗ 0.9CM) and left adnexal sac (3.1 ∗ 2.3 cm).

### 2.4 Diagnosis and treatment process

**Figure 2:**
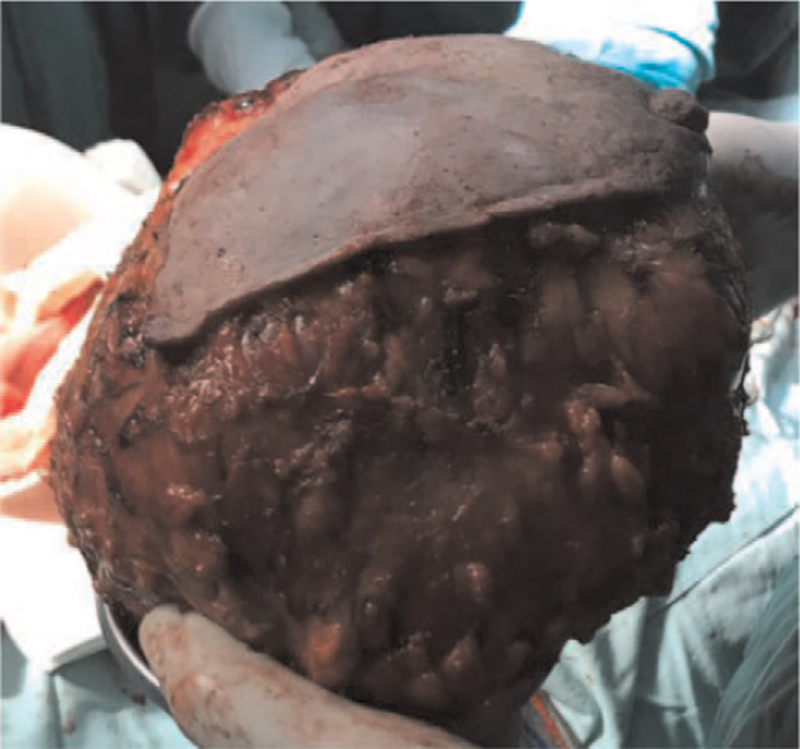
The size of tumor resected specimen is 23 × 20 × 11cm and the spindle skin is 18 × 13cm.

**Figure 3:**
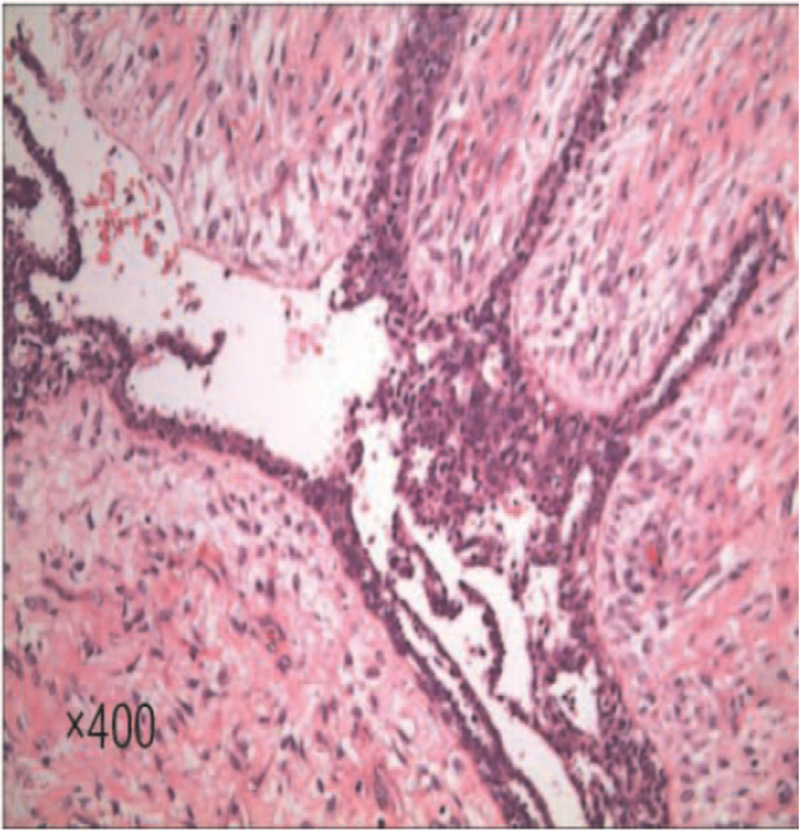
Postoperative pathology showed that the tumor tissue was composed of epithelial or stromal components, with obvious hyperplasia of stromal components, mild atypia, mitotic figure and lobulated structure.

After admission, the patient suffered from dizziness, palpitation, and weakness several times, accompanied by disturbance of consciousness, which was relieved after oral glucose or intravenous infusion, and the glucose levels decreased significantly. Combined with the glucose tolerance test, it was suggested that hypoglycemia was not caused by insulin secretion. Biopsy of the right breast mass showed fibroepithelial tumor with active growth of stromal cells. Right mastectomy was performed under general anesthesia. The surgery was successful, and the postoperative recovery was good (Fig. 2). Postoperative pathology showed borderline phyllodes tumor (right breast), nipple (-), skin (-), basal (-), and stromal cell proliferation was obvious, with mild atypia and mitotic figures (Fig. 3). Immunohistochemistry indicated Er-α in the average staining intensity, Ki67 positive cells accounted for about 2%, p53 positive cells accounted for less than 1%, E-cadherin (+), p120 membrane (+), CD34 vascular (+), CD117 (+), and smooth muscle actin (+). On the third day after surgery, fasting blood glucose was 4.75 mmol / L, fasting insulin was 10.51 mu/L - all in the normal range. The patient came to our hospital for re-examination every 6 months. After 3 years of follow-up, hypoglycemia did not recur, and she was in good condition.

## 3 Discussion

This is a case of hypoglycemia caused by phyllodes tumor of breast, which belongs to non-islet cell tumor hypoglycemia. In 1929, Nadler et al^[2]^ reported for the first time a case of liver cancer-related hypoglycemia. Since then, there have been different types of non-islet cell tumor cases reported, all of which have hypoglycemic symptoms.

Xu et al^[1]^ reported the statistics of NICTH-related literature of data collected in 10 years. Out of a total of 61 cases, 91.8% of cases reported hypoglymeia, and only 2 cases was caused by breast cancer, accounting for 3.3%. In 2014, Bodnar et al^[3]^ reviewed the literature from January 1988 to August 2013, where 98 cases of NICTH were reported. Therefore, clinically, NICTH is a rare paraneoplastic syndrome, especially caused by breast cancer. When the tumor is a fibroma of the chest, it is called Doege Potter syndrome.^[4,5]^ NICTH can occur at any age, mostly in middle-aged and elderly individuals. Tumor growth in this condition is slow, but it is usually large, mostly occurs in malignant tumors, and the majority of tumors originate from mesenchymal and epithelial tissues.

The possible mechanisms of NICTH are as follows:

1. tumor tissue growth and energy consumption increase;
2. the tumor increases the insulin stimulating hormone or decreases the insulin inhibiting hormone;
3. gluconeogenesis pathway disorder;
4. tumors produce a large number of substances with insulin activity, which can produce insulin effect,^[6,7]^ which was initially attributed to the increase in tumor size and glucose utilization.

The real mechanism of hypoglycemia caused by non-islet cell tumors was still unclear in the 1970s and the early 1980s, when circulating insulin-like peptides were described.^[8,9]^ In 1988, Daughaday et al^[10]^ studied abnormal (incompletely processed) IGF-II, and finally described high molecular weight or “large” IGF-II,^[11]^ which has strong insulin-like activity and leads to hypoglycemia. IGF-II is homologous with insulin, which can cause hypoglycemia by inhibiting glucose output from the liver and enhancing glucose uptake from skeletal muscle; insulin-like growth factor II activates insulin receptor, promotes the continuous utilization of glucose in skeletal muscle, and inhibits the release of free fatty acids from adipocytes, which also leads to the inhibition of glucose release, glycogenolysis, gluconeogenesis, and ketogenesis in the liver.^[12]^ In addition, IGF-II inhibited the release of glucagon and growth hormone, which in turn amplified the degree of hypoglycemia in NICTH.^[13]^

As early as 1988, Daughaday et al^[10]^ showed that IGF-II mRNA is highly expressed in tumor cells. The IGF-II gene is located in 5 bands of short arm 1 of chromosome 11. Under normal circumstances, the IGF-II gene only expresses the paternal allele, that is, the maternal imprinted gene, as well as 2 tumor suppressor genes, H19 and p57k1p2, in this chromosomal region, which may be caused by the deletion of imprinted genes and the non-expression of tumor suppressor genes.^[14]^ IGF-II gene transcription and translation products are 180 amino acid residues, namely pre-IGF-II, including 24 amino acid peptide N-terminal, 67 amino acid mature IGF-II, and 89 amino acid C-terminal extension, called E-domain. The E-domain was cleaved to form large IGF-II, which was then hydrolyzed to IGF-II. Generally, it is a single-chain polypeptide composed of 67 amino acids, with a molecular weight of 7.5 × 10^5^, but 10 to 20 × 10^5^ in NICTH, called large IGF-II. The biological activity of IGF-II is 10 times higher than that of IGF-II.^[15–17]^ Under normal conditions, IGF-II binds IGF binding protein 3 to form a binary complex (50–60 × 10^5^), and then combines with an unstable subunit to form a ternary complex (140–150 × 10^5^). In normal human serum, binary complexes accounted for 20% to 30%, ternary complex about 70% to 80%, and free components account for less than 1%. In NICTH, the spatial phase of large IGF-II changes and cannot be combined into ternary complexes. Therefore, approximately 80% exist in the form of binary complexes.^[18]^ Compared with the ternary complex, the binary complex has a smaller molecular weight, shorter half-life, and faster metabolism. It is easier to pass through the vascular endothelium, enter the tissue, bind with the receptor, and play a role in hypoglycemic insulin-like activity.^[19,20]^

There is no international standard for the diagnosis of NICTH, in view of the fact that most of the previous reports^[21]^ have the following characteristics: ① recurrent hypoglycemia usually occurs during fasting and at night; ② the tumor volume is usually large,^[22]^ which is more common in middle-aged and elderly people; ③ the levels of insulin and C-peptide were low; and ④ IGF-II levels may or may not increase. Large IGF-II levels increase and IGF-I levels decrease, but the ratio of IGF-II: IGF-I will increase, often close to or more than 10:1;^[23]^⑤ Other causes of hypoglycemia were excluded. Other causes of hypoglycemia were excluded based on the hypoglycemic diagnosis procedure.^[24,25]^

Timely intravenous or oral glucose supplementation can relieve the symptoms of hypoglycemia and prevent irreversible brain damage caused by severe hypoglycemia. The key to the treatment of NICTH is removal of the primary tumor.^[23]^

NICTH is rare, especially in breast cancer, and should be considered in patients with recurrent hypoglycemia, after excluding other insulinomas and pancreatic origin. Clinicians should improve their understanding of this condition for early diagnosis and treatment. Complete resection of the primarytumor is the most effective treatment for NICTH.

## Author contributions

**Conceptualization:** Zhibing Zhou.

**Data curation:** Zhibing Zhou, Wensong Wei.

**Investigation:** Qihua Jiang.

**Methodology:** Jianhong Tu.

**Resources:** Zhibing Zhou.

**Supervision:** Jianhong Tu, Qihua Jiang.

**Writing – original draft:** Zhibing Zhou.

**Writing – review & editing:** Zhibing Zhou, Wensong Wei.

## References

[1] Xu Tao Chen Xian Dai Zhenzhen. One case of non islet cell tumor hypoglycemia caused by bladder cancer and literature review. Chin General Pract 2018;16:185–7.

[2] Nadler WA Wolfer JH. Hepatogenic hypoglycemia associated with primary liver cell carcinoma. Arch Intern Med 1929;44:700–10.

[3] Bodnar TW Acev Ed OMJ Massimo P. Management of non-islet-cell tumor hypoglycemia: a clinical review. J Clin Endocrinol Metab 2014;03. 10.1210/jc.2013-3382 PMC5393479 24423303

[4] Reuvers J Dorp MV Schil PV. Solitary fibrous tumor of the pleura with associated Doege-Potter syndrome. Acta Chirurgica Belgica 2016;386–7. 27376978 10.1080/00015458.2016.1171079

[5] Qin Yao Ke Nengwen Yin Wanhong. A case of pleura malignant solitary fibrous tumor with Doege Potter syndrome. West China Med 2016;138–9.

[6] Doege KW. Fibrosarcoma of the mediastinum. Ann Surg 1930;92:955–60. 17866430 PMC1398259

[7] Marks V Samols E. Hypoglycaemia of non-endocrine origin (non-islet cell tumours). Proc R Soc Med 1966;59:338–40. 5937685 10.1177/003591576605900412 PMC1900615

[8] Klara M Ronald KC Jesse R. Hypoglycemia in association with extrapancreatic tumors: demonstration of elevated plasma nsila-s by a new radioreceptor assay. J Clin Endocrinol Metab 1974;931–4. 4823931 10.1210/jcem-38-5-931

[9] Zapf J Walter H Froesch ER. Radioimmunological determination of insulinlike growth factors I and II in normal subjects and in patients with growth disorders and extrapancreatic tumor hypoglycemia. J Clin Investig 1981;68:1321. 7028787 10.1172/JCI110379 PMC370928

[10] Daughaday WH Emanuele MA Brooks MH. Synthesis and secretion of insulin-like growth factor II by a leiomyosarcoma with associated hypoglycemia. N Engl J Med 1988;22:1434–40. 10.1056/NEJM198812013192202 3185662

[11] Zapf J Futo E Peter M Froesch ER. Can “big” insulin-like growth factor II in serum of tumor patients account for the development of extrapancreatic tumor hypoglycemia? J Clin Invest 1992;90:2574–84. 1281841 10.1172/JCI116152 PMC443417

[12] Dynkevich Y Rother KI Whitford I. Tumors, IGF-2 and hypoglycemia: insights from the clinic, the laboratory and the historical archive. Endocr Rev 2013;34:798–826. 23671155 10.1210/er.2012-1033

[13] LeRoith D Roberts CT. The insulin-like growth factor system and cancer. Cancer Lett 2003;195:127–37. 12767520 10.1016/s0304-3835(03)00159-9

[14] Silveira L F. Growth hormone therapy for non-islet cell tumor hypoglycemia. Am J Med 2002. 10.1016/s0002-9343(02)01180-4 12208393

[15] Yang CQ Zhan X Hu X. The expression and characterization of human recombinant proinsulin-like growth factor II and a mutant that is defective in the O-glycosylation of its E domain. Endocrinology 1996;2766–73. 8770896 10.1210/endo.137.7.8770896

[16] Roith DL. Seminars in medicine of the Beth Israel Deaconess Medical Center. Insulin-like growth factors. N Engl J Med 1997;336:633–40. 9032050 10.1056/NEJM199702273360907

[17] Izumi F Akira A Tomoko N. Levels of glucose-regulatory hormones in patients with non-islet cell tumor hypoglycemia: including a review of the literature. Endocr J 2017;64:719–26. 28529277 10.1507/endocrj.EJ17-0072

[18] Bond J. Binding characteristics of pro-insulin-like growth factor-II from cancer patients: binary and ternary complex formation with IGF binding proteins-1 to -6. J Endocrinol 2000. 10.1677/joe.0.1650253 10810289

[19] Guler HP Zapf J Schmid C. Insulin-like growth factors I and II in healthy man. Estimations of half-lives and production rates. Acta Endocrinol 1989;121:753–8. 10.1530/acta.0.1210753 2558477

[20] Frystyk J Skjaerbaek C Zapf J. Increased levels of circulating free insulin-like growth factors in patients with non-islet cell tumour hypoglycaemia. Diabetologia 1998;41:589–94. 9628278 10.1007/s001250050951

[21] Chen Xuanqing Zhang Qiu. Research progress of non-islet cell tumor hypoglycemia. Med Rev 2013.

[22] Fukuda I Hizuka N Ishikawa Y. Clinical features of insulin-like growth factor-II producing non-islet-cell tumor hypoglycemia. Growth Hormone & Igf Research Official Journal of the Growth Hormone Research Society & the International Igf Research Society 2006;16:211–6. 10.1016/j.ghir.2006.05.003 16860583

[23] Bodnar TW. Management of non-islet-cell tumor hypoglycemia: a clinical review. J Clin Endocrinol Metab 2014. 10.1210/jc.2013-3382 PMC5393479 24423303

[24] Iglesias P Diez JJ. Management of endocrine disease: a clinical update on tumor-induced hypoglycemia. Eur J Endocrinol 2014;170:147–57. 10.1530/EJE-13-1012 24459236

[25] Lu Jieli Liu Jianmin Fang Wenqiang. Hypoglycemia caused by non-islet cell tumor: a case report and literature review. Chin J Endocrinol Metab 2016;32:330–4.

